# Supplementary material for: A variational approach to parameter estimation in ordinary differential equations
Source: BMC Syst Biol. 2012 Aug 14;6:99. doi: 10.1186/1752-0509-6-99 (PMC3534504; doi:10.1186/1752-0509-6-99)
Supplement: Additional file 1 — Supplement: A Variational Approach to Parameter Estimation in Ordinary Differential Equations. [file 1752-0509-6-99-S1.pdf]

Supplement: A Variational Approach to Parameter Estimation in Ordinary Differential Equations

Daniel Kaschek

July 4, 2012

Contents

1 Parameter estimation in ODE systems with inputs **1**

2 The Gâteaux differential of $\chi^2[x]$ **2**

3 Discretization of the variational problem **4**

4 Analytical solution with strict boundary condition **5**

5 Analytical solution with relaxed boundary condition **6**

6 Working with noise and observation function **7**

1 Parameter estimation in ODE systems with inputs

Let

$$\dot{y} = f(y, [x], p) \tag{1}$$

be an ODE for components $y \in \mathbb{R}^N$ with continuous input function $[x] \in C^0(\mathbb{R}, \mathbb{R}^M)$. The system depends on dynamic parameters $p \in \mathbb{R}^K$ and on initial conditions $y(t = 0)$ which are combined to $P = (p, y(t = 0))$. The solution to eq. (1) is denoted by $y_P[x]$ and is a differentiable function of time t. Let us assume that the components y are measured at time points t_i denoted by y_i. For fixed $[x]$ the χ^2 function

$$\chi^2_{\text{red}}(P) = \sum_i \|y_P[x](t_i) - y_i\|^2 =: \sum_i \|\text{res}_y(t_i)\|^2 \tag{2}$$

is a function of parameters P and the optimal parameters $\hat{P}$ minimizing eq. (2) are approached by an optimization method. For varying input function $[x]$, the solution trajectory $y_P[x]$ and the optimal parameters $\hat{P}$ vary as well and depend on the entire course of $[x]$. The question is, what is the optimal choice for the input function resulting in the lowest total χ^2 value.

To this purpose, we are going to deal with variational calculus. First, the functional of optimal parameters $\hat{P}[x]$ is characterized by

$$\forall k: \left.\frac{\partial \chi^2_{\text{red}}}{\partial P_k}\right|_{\hat{P}[x]} = \frac{\partial}{\partial P_k} \sum_i \|y_P[x](t_i) - y_i\|^2 \Bigg|_{P=\hat{P}[x]} = 0. \tag{3}$$

Thereby, the total χ^2 becomes a functional of $[x]$:

$$\chi^2[x] = \sum_i \|y_{\hat{P}[x]}[x](t_i) - y_i\|^2 + \|x(t_i) - x_i\|^2 \tag{4}$$

The second term introduces an objective function for deviations from the x-measurements. The Gâteaux differential of $\chi^2[x]$ is defined as

$$\mathrm{d}\chi^2([x], h) := \left.\frac{\mathrm{d}\chi^2[x + \epsilon h]}{\mathrm{d}\epsilon}\right|_0 = 2\sum_i \Big\langle \mathrm{res}_y(t_i),\ \mathrm{d}y_{\hat{P}}([x], h)(t_i)\Big\rangle + \Big\langle \mathrm{res}_x(t_i), h(t_i)\Big\rangle. \tag{5}$$

which is homogeneous, i.e. $\forall \alpha \in \mathbb{R} :\ \mathrm{d}\chi^2([x], \alpha h) = \alpha\ \mathrm{d}\chi^2([x], h)$ but in general it is whether continuous nor is it linear. If $\mathrm{d}\chi^2([x], h)$ is continuous in h,

$$\forall h :\ \ \mathrm{d}\chi^2([\hat{x}], h) = 0 \tag{6}$$

is a necessary condition for an extremal solution $[\hat{x}]$. If $\mathrm{d}\chi^2([x], h) = \delta\chi^2[x]h$ is in addition linear in h then $\delta\chi^2[x]$ is called the *first variation* of χ^2 and the extremal condition becomes $\delta\chi^2[\hat{x}] = 0$. From the practical point of view, linearity is a major feature for other reasons. Assume, we want to find an approximate extremal solution $[\hat{x}]$ in a finite dimensional function space X_0 defined by a finite basis $\{h_1, \dots, h_n\}$. Then by linearity,

$$\forall h \in X_0 :\ \chi^2[x + h] \doteq \chi^2[x] + \sum_k \alpha_k \delta\chi^2[x]h_k \tag{7}$$

$$= \chi^2[x] + \big\langle \alpha, \nabla\chi^2[x]\big\rangle \tag{8}$$

where $\nabla\chi^2[x]_k := \delta\chi^2[x]h_k$. I.e. $[x]$ is approximately extremal only if $\nabla\chi^2[x] = 0$ and starting from a non-extremal $[x]$, $h = \sum_k \nabla\chi^2[x]_k h_k$ indicates the direction to the extremum. In the framework of numerical optimization theory, linearity is absolutely necessary.

With these considerations in mind the Gâteaux differential of χ^2 is analyzed. The following section will show linearity as well as continuity.

2 The Gâteaux differential of $\chi^2[x]$

The differential of χ^2 depends on the differential of the trajectory $y_{\hat{P}[x]}[x]$. Since every trajectory satisfies the differential equation $\dot{y}_{\hat{P}[x]}[x] = f(y_{\hat{P}[x]}[x], [x], \hat{P}[x])$, we have

$$\frac{d}{dt}\ \mathrm{d}y_{\hat{P}}(x, h) = \mathsf{D}_y f\ \mathrm{d}y_{\hat{P}}(x, h) + \mathsf{D}_x f\ h + \mathsf{D}_P f\ \mathrm{d}\hat{P}(x, h), \tag{9}$$

where $\mathsf{D}_y f$ denotes the Jacobian of f with respect to y and analogously $\mathsf{D}_x f$ and $\mathsf{D}_P f$. The optimal choice of parameters $\hat{P}[x]$ is defined by eq. (3) and explicitly reads:

$$\left.\frac{\partial\chi^2_{\mathrm{red}}}{\partial P_k}\right|_{\hat{P}} = 2\sum_i \Big\langle \mathrm{res}_y(t_i), \left.\frac{\partial y_P[x]}{\partial P_k}\right|_{\hat{P}}(t_i)\Big\rangle = 0 \equiv \Phi_k(\hat{P}[x], [x]). \tag{10}$$

Consequently, the Gâteaux differential of Φ restricted to $(\hat{P}[x], [x])$ vanishes and we get

$$\mathrm{d}\Phi(x, h) = \mathsf{D}_P\Phi|_{\hat{P}[x]}\ \mathrm{d}\hat{P}(x, h) + \partial\Phi(x, h) = 0 \tag{11}$$

$$\Rightarrow \quad \mathrm{d}\hat{P}(x, h) = -C\,\partial\Phi(x, h) \tag{12}$$

with $\partial\Phi(x,h) := \frac{\mathrm{d}\Phi(\hat{P}[x],[x+\epsilon h])}{\mathrm{d}\epsilon}$ and $\mathsf{D}_P\Phi|_{\hat{P}[x]}$ is the inverse of the covariance matrix C. In the following, differentials denoted by ∂ mean that $P = \hat{P}[x]$ is fixed. By eq. (10)

$$\partial\Phi_k(x,h) = 2\sum_i \left\langle \partial y_{\hat{P}}(x,h)(t_i), \left.\frac{\partial y_P[x]}{\partial P_k}\right|_{\hat{P}}(t_i)\right\rangle + \left\langle \mathrm{res}_y(t_i), \partial \left.\frac{\partial y_P}{\partial P_k}\right|_{\hat{P}}(x,h)(t_i)\right\rangle. \tag{13}$$

The unknown functions are expressed by their corresponding differential equations, hence

$$\frac{d}{dt}\partial y_{\hat{P}}(x,h) = \mathsf{D}_y f\ \partial y_{\hat{P}}(x,h) + \mathsf{D}_x f\ h \tag{14}$$

$$\frac{d}{dt}\frac{\partial y_P[x]}{\partial P_k} = \mathsf{D}_y f\ \frac{\partial y_P[x]}{\partial P_k} + \frac{\partial f}{\partial P_k} \tag{15}$$

$$\frac{d}{dt}\left(\partial \left.\frac{\partial y_P}{\partial P_k}\right|_{\hat{P}}\right)(x,h) = \partial\left(\mathsf{D}_y f\ \left.\frac{\partial y_P[x]}{\partial P_k}\right|_{\hat{P}} + \left.\frac{\partial f}{\partial P_k}\right|_{\hat{P}}\right)(x,h) \tag{16}$$

$$= (\partial\mathsf{D}_y f)(x,h)\ \left.\frac{\partial y_P[x]}{\partial P_k}\right|_{\hat{P}} + \mathsf{D}_y f\left(\partial \left.\frac{\partial y_P}{\partial P_k}\right|_{\hat{P}}\right)(x,h) + \left(\partial \left.\frac{\partial f}{\partial P_k}\right|_{\hat{P}}\right)(x,h). \tag{17}$$

Eq. (15) has initial condition zero for dynamical parameters P_k and one for the corresponding component of y if P_k is an initial concentration. Eqs. (14, 17) have zero initial condition which is due to the fact that both $y_P(t=0)$ and $\frac{\partial y_P}{\partial P_k}(t=0)$ are fixed by the corresponding parameters P_k of initial concentrations no matter what x is. Finally, the unknown differentials $\partial\mathsf{D}_y f$ and $\partial\frac{\partial f}{\partial P_k}$ can be evaluated and stated explicitly:

$$(\partial\mathsf{D}_y f)(x,h) = \mathsf{D}_y^2 f\ \partial y_{\hat{P}}(x,h) + \mathsf{D}_y\mathsf{D}_x f\ h \tag{18}$$

$$\left(\partial \left.\frac{\partial f}{\partial P_k}\right|_{\hat{P}}\right)(x,h) = \frac{\partial}{\partial P_k}\mathsf{D}_y f\ \partial y_{\hat{P}}(x,h) + \frac{\partial}{\partial P_k}\mathsf{D}_x f\ h. \tag{19}$$

At this point, $\mathrm{d}\chi^2(x,h)$ is completely defined by derivatives of f, the covariance matrix C of the reduced parameter estimation problem and the solutions to the differential equations (14-17). These differential equations have the special feature that they are of the form

$$\dot{y}(t) = M(t)y(t) + h(t) \tag{20}$$

with $M : \mathbb{R} \to \mathrm{Hom}(\mathbb{R}^n)$. The solution to this equation is explicitly given by

$$y(t) = \Phi(t)\left(\int_0^t \Phi^{-1}(\tau)h(\tau)\ \mathrm{d}\tau + y_0\right) \tag{21}$$

$$=: \mathcal{J}[M,h](t) + \Phi(t)y_0 \tag{22}$$

with $\Phi(t) := \exp\left(\int_0^t M(\tau)\ \mathrm{d}\tau\right)$. This explicit formula is the key to the linearity of $\mathrm{d}\chi^2(x,h)$. By the linearity of the integral,

$$\mathcal{J}[M,\alpha h + \beta g] = \alpha\mathcal{J}[M,h] + \beta\mathcal{J}[M,g] \tag{23}$$

is linear in the second argument. Furthermore,

$$\partial y_{\hat{P}}(x,h) = \mathcal{J}[\mathsf{D}_y f, \mathsf{D}_x f h] \tag{24}$$

is linear in h and thereby also eqs. (18) and (19) are linear in h. Consequently,

$$\left(\partial \left.\frac{\partial y_{\hat{P}}}{\partial P_k}\right|_{\hat{P}}\right) = \mathcal{J}\left[\mathsf{D}_y f, (\partial\mathsf{D}_y f)\ \left.\frac{\partial y_P[x]}{\partial P_k}\right|_{\hat{P}}(x,h) + \left(\partial \left.\frac{\partial f}{\partial P_k}\right|_{\hat{P}}\right)(x,h)\right] \tag{25}$$

is linear in h as well as $\partial\Phi_k(x,h)$, $\mathrm{d}\hat{P}(x,h)$, $\mathrm{d}y_{\hat{P}}(x,h)$ and finally $\mathrm{d}\chi^2(x,h)$ so that we come to the important result:

$$\mathrm{d}\chi^2(x,h) = \delta\chi^2(x)\,h \tag{26}$$

is a linear functional. Due to the explicit solutions of eqs. (14-17), $\mathrm{d}\chi^2(x,h)$ is also continuous for appropriate f.

3 Discretization of the variational problem

One possibility to approach $[\hat{x}]$ is discretization of the function space from which $[\hat{x}]$ originates. An adequate finite dimensional subspace $X_0 \subset X$ has to be chosen. Inspired by Fourier or Laplace transformations, bases of X_0 could be

$$h_k(t) = \begin{cases} \sin(k\omega t) & ,k = 1,3,5,\ldots \\ \cos(k\omega t) & ,k = 2,4,6,\ldots \end{cases} \tag{27}$$

or

$$h_k(t) = e^{-k\gamma t} \tag{28}$$

and k should be bounded.

Another possibility would be to choose smooth spline functions. Let $x_S(t; x_1, \ldots, x_n, \lambda)$ be the smooth spline function defined by nodes x_i at times t_i, i.e. x_S is the smooth spline through the measurement points of x. Then

$$h_k(t) = \frac{\partial x_S(t; x_1, \ldots, x_n, \lambda)}{\partial x_k} \tag{29}$$

defines a local basis of a function space of smooth spline functions. This approach is closely connected to the estimation problem by the objective function

$$\chi^2(P, x_1, \ldots, x_n) = \sum_i \|y_P[x_S](t_i) - y_i\|^2 + \|x_S(t_i; x_1, \ldots, x_n) - x_i\|^2. \tag{30}$$

When computing $\frac{\partial \chi^2}{\partial x_k}$, derivatives of x_S and $y_P[x_S]$ have to be computed:

$$\frac{\partial x_S}{\partial x_k}(t_i) = \left.\frac{d}{d\epsilon}\right|_{\epsilon=0} x_S(t_i, x_k + \epsilon) \tag{31}$$

$$\frac{\partial y_P}{\partial x_k}(t_i) = \left.\frac{d}{d\epsilon}\right|_{\epsilon=0} y_P[x_S(t_i; x_k + \epsilon)] \tag{32}$$

$$= \left.\frac{d}{d\epsilon}\right|_{\epsilon=0} y_P[x_S(t_i; x_k) + \epsilon \frac{\partial x_S}{\partial x_k}(t_i)] \tag{33}$$

$$= \delta y_P(x_S) \frac{\partial x_S}{\partial x_k}(t_i) \tag{34}$$

$$= \delta y_P(x_S) h_k(t_i). \tag{35}$$

In other words

$$(\nabla\chi^2[x_S])_k := \delta\chi^2(x_S) h_k = \frac{\partial \chi^2(P, x_1, \ldots, x_n)}{\partial x_k}. \tag{36}$$

Conclusion The functional of the first variation applied to the basis functions coincides with the gradient which is usually employed by the optimizer to optimize the parameters $x_1, \dots, x_n$.

4 Analytical solution with strict boundary condition

If the differential equation $\dot{y} = f(y, x)$ is linear with respect to x, a differential equation for $x(t)$ can be derived from the condition of vanishing first variation. Since in $\delta\chi^2(x)h$ the variation $h(t)$ occurs one time under the integral and one time not, this has to be resolved by partial integration. Therefore, res_y and res_x as defined in eq. (2) for discrete time points need to become continuous functions of t. This can be realized by interpolation splines

$$S_x(t) = \mathsf{spline}(x_1, \dots, x_n)(t) \tag{37}$$

$$S_y(t) = \mathsf{spline}(y_1, \dots, x_y)(t). \tag{38}$$

Thereby, $y(t) - S_y(t)$ becomes a function of t with $y(t_i) - S_y(t_i) = y(t_i) - y_i = \mathrm{res}_y(t_i)$ and analogously for x. Another issue however is not resolvable. The variation $\delta\hat{P}(x)h$ does not depend on t at all. Therefore, h cannot be extracted from $\delta\hat{P}(x)h$ by means of differentiation or partial integration. The problem has to be reformulated as finding the optimal $x(t)$ for given data **and** given parameters. Thereby, we have to minimize

$$\chi^2[x|P] = \int_0^T \left((y(t) - S_y(t))^2 + (x(t) - S_x(t))^2\right)\, \mathrm{d}t \tag{39}$$

$$= \int_0^T \left(\mathrm{res}_x^2 + \mathrm{res}_y^2\right)\, \mathrm{d}t. \tag{40}$$

For the first variation we have

$$\frac{1}{2}\delta\chi^2(x|P)h = \int_0^T \left\langle \mathrm{res}_y(t), \Phi(t) \int_0^t \Phi^{-1}(\tau)\mathsf{D}_x f(\tau)h(\tau)\, \mathrm{d}\tau \right\rangle + \left\langle \mathrm{res}_x(t), h(t) \right\rangle\, \mathrm{d}t \tag{41}$$

and by partial integration

$$= \left[\int_0^t \Phi(t)^* \mathrm{res}_y(t)\, \mathrm{d}t, \int_0^t \Phi^{-1}(t)\mathsf{D}_x f(t)h(t)\, \mathrm{d}t\right]_0^T \tag{42}$$

$$- \int_0^T \left\langle \int_0^t \Phi(\tau)^* \mathrm{res}_y(\tau)\, \mathrm{d}\tau, \Phi^{-1}(t)\mathsf{D}_x f(t)h(t) \right\rangle - \left\langle \mathrm{res}_x(t), h(t) \right\rangle\, \mathrm{d}t. \tag{43}$$

In order to find a unique solution, one requires $\int_0^T \Phi^{-1}(t)D_x f(t)h(t)\, \mathrm{d}t$ to vanish. This means that only variations h are taken into consideration that leave initial and end point of $y(t)$ untouched. Since eq. (43) has to be zero for all such h, the equation

$$\mathsf{D}_x f(t)^* \underbrace{\Phi^{-1}(t)^* \int_0^t \Phi(\tau)^* \mathrm{res}_y(\tau)\, \mathrm{d}\tau}_{=:u(t)} - \mathrm{res}_x(t) = 0 \tag{44}$$

has to be solved. This integral equation can be transformed into an equivalent system of differential equations. Therefor, we use that $\Phi = \exp\left(\int_0^t \mathsf{D}_y f(\tau)\, \mathrm{d}\tau\right)$ is a fundamental system for $\dot{\phi} = \mathsf{D}_y f \phi$ and consequently, Φ^{-1} is a fundamental system for $\dot{\phi} = -\mathsf{D}_y f \phi$. From that, eq. (46)

follows. For the time derivative of x we distinguish different cases. If $\mathsf{D}_x f = const.$, it is not affacted by the time derivative leading to eq. (47). The entire ODE system then reads:

$$\dot{y} = f(y, x) \tag{45}$$

$$\dot{u} = -\mathsf{D}_y f(y, x)^* u + y - S_y(t) \tag{46}$$

$$\dot{x} = \dot{S}_x(t) + \mathsf{D}_x f^* \dot{u}. \tag{47}$$

If $\mathsf{D}_x f$ is not constant, its time derivative is

$$\frac{\mathrm{d}}{\mathrm{d}t}\mathsf{D}_x f = \mathsf{D}_x \frac{\mathrm{d}f}{\mathrm{d}t} = \mathsf{D}_x \left(\mathsf{D}_y f \dot{y} + \mathsf{D}_x f \dot{x}\right) \tag{48}$$

and we distinguish the case $\mathsf{D}_x^2 f = 0$ where

$$\dot{x} = \dot{S}_x(t) + \dot{y}^* \mathsf{D}_y \mathsf{D}_x f(y, x)^* u + \mathsf{D}_x f(y, x)^* \dot{u}. \tag{49}$$

and $\mathsf{D}_x^2 f \neq 0$ where

$$\dot{x} = \dot{x}^* \mathsf{D}_x^2 f(y, x)^* u + H(y, x, u, t) \tag{50}$$

with some function H that depends on y, x, u and t but not on $\dot{x}$. In this case, it is unclear whether $\mathbb{1} - \mathsf{D}_x^2 f(y, x)^* u$ is invertible or not. Therefore, a unique solution for (y, u, x) only exists if $\mathbb{1} - \mathsf{D}_x^2 f(y, x)^* u$ is invertible alongside the whole trajectory.

5 Analytical solution with relaxed boundary condition

When optimizing $\chi^2[\hat{x}(P), P]$ with respect to P, the resulting $\hat{x}(\hat{P})$ looks very promising and follows the data points closely. Parameter estimation with repeated noise realizations reveals, that $\hat{P}$ is an unbiased estimator with a variance that is about the variance of the original estimation problem. However, the main problem is that starting with parameters P that are slightly different from $\hat{P}$,

However, the trajectory $x(t)$ obtained by the above differential equation for parameters P differing slightly from $\hat{P}$ may be far away from the data points. In most cases it does not seem like an optimal solution at all. Though, tests with such optimal solutions $\hat{x}$ reveal that indeed, for any h obeying the boundary condition (42), $\chi^2[\hat{x} + \epsilon h] > \chi^2[\hat{x}]$ for $\epsilon \neq 0$ and ϵ in a neighborhood of zero. This problem is resolved by relaxing the boundary condition.

For real valued functions f and g we have the following general equation which can be shown by partial integration:

$$\int_0^{t'} f(t) \left(\int_0^t g(\tau)\ \mathrm{d}\tau\right)\ \mathrm{d}t = \int_0^{t'} \left(\int_t^{t'} f(\tau)\ \mathrm{d}\tau\right) g(t)\ \mathrm{d}t \tag{51}$$

Applying this formula to (41), we get

$$\frac{1}{2}\delta\chi^2(x)h = \int_0^T \Big\langle \int_t^T \Phi(\tau)^* \mathrm{res}_y(\tau)\ \mathrm{d}\tau, \Phi^{-1}(t)\mathsf{D}_x f(t) h(t)\Big\rangle + \Big\langle \mathrm{res}_x(t), h(t)\Big\rangle\ \mathrm{d}t \tag{52}$$

and therefore

$$\mathsf{D}_x f(t)^* \underbrace{\Phi^{-1}(t)^* \int_t^T \Phi^*(\tau)\mathrm{res}_y(\tau)\ \mathrm{d}\tau}_{=:u(t)} + \mathrm{res}_x(t) \stackrel{!}{=} 0 \tag{53}$$

This leads to the very similar system of differential equations

$$\dot{y} = f(y, x) \tag{54}$$
$$\dot{u} = -\mathsf{D}_y f(y, x)^* u - (y - S_y(t)) \tag{55}$$
$$\dot{x} = \dot{S}_x(t) - \mathsf{D}_x f^* \dot{u} - \frac{\mathrm{d}}{\mathrm{d}t}\mathsf{D}_x f(t)^* u. \tag{56}$$

with the only difference that the initial condition of x and u already depend on the solution of the ODE. In contrast, the final conditions $u(T) = 0$ and $x(T) = S_x(T)$ follow directly from eq. (53). Hence, the price to pay for releasing the boundary condition of h is that the initial value problem becomes a two way boundary value problem, which is numerically demanding.

6 Working with noise and observation function

The basic objective function used so far is the unweighted sum of squares. In the context of parameter estimation in chemical and biochemical reaction networks, some components may be measured more precisely than others. Several of the components might be unobserved or only linear combinations might be observable. In order to deal with these issues, the formalism needs to be adjusted.

Let G be an observation function so that

$$\tilde{z}(t) = G(y(t), x(t)). \tag{57}$$

In order to deal with the approach derived above, it is necessary that G separates into two parts G_x and G_y, so that

$$\tilde{y}(t) = G_y(y(t)) \tag{58}$$
$$\tilde{x}(t) = G_x(x(t)). \tag{59}$$

In addition, we assume noise functions $\sigma_{\tilde{x}}$ and $\sigma_{\tilde{y}}$ for the data. Then, eq. (53) becomes

$$\mathsf{D}_x f^* \big(\Phi^{-1}\big)^* \int_t^T \Phi^* \mathsf{D}_y G_y^* \frac{\tilde{y} - S_{\tilde{y}}}{\sigma_{\tilde{y}}^2} \, \mathrm{d}\tau + \mathsf{D}_x G_x^* \frac{\tilde{x} - S_{\tilde{x}}}{\sigma_{\tilde{x}}^2} = 0. \tag{60}$$

Like above, linearity in the input is sufficient to guarantee a solution. In this case this means that G_x is expected to be linear. The ODE system to solve is

$$\dot{y} = f(y, x) \tag{61}$$
$$\dot{u} = -\mathsf{D}_y f(y, x)^* u - \mathsf{D}_y G_y^* \frac{G_y(y) - S_{\tilde{y}}}{\sigma_{\tilde{y}}^2} \tag{62}$$

with $x = G_x^{-1}\big(S_{\tilde{x}} - \sigma_{\tilde{x}}^2 \big(\mathsf{D}_x G_x^*\big)^{-1} \mathsf{D}_x f^* u\big)$.

Remark 6.1 (Observational function for the input) Since there are strong requirements to G_x, it is most convenient to make a transformation of the data beforehand to circumvent dealing with $\tilde{x}$. The same works for y. If G_y has not full rank meaning that some components are unobserved, these components do not contribute to $\dot{u}$. Say $G_y = \mathsf{diag}(1, \ldots, 1, 0, \ldots, 0)$. In other cases it might be necessary do deal at least with G_y, e.g. if G_y is a nonlinear transformation which produces normally distributed data $\tilde{y}$ whereas y itself is not normally distributed.

Remark 6.2 (Noise) If noise in the input measurements is considered, the condition for constructing x uniquely from u is that $\mathsf{D}_x^2 f^* u + \frac{1}{\sigma_x^2}\mathbb{1}$ be invertible, analogously to eq. (50). Or equivalently

$$\sigma_x^2 \mathsf{D}_x^2 f^* u + \mathbb{1} \text{ be invertible.} \tag{63}$$

This is the case if the spectral radius (largest absolute eigenvalue)

$$\rho(\sigma_x^2 \mathsf{D}_x^2 f^*) < 1 \tag{64}$$

is smaller than 1. That can be achieved if the curvature $\mathsf{D}_x^2 f$ is bounded over all feasible solution points. I.e. let $U \subset \mathbb{R}^N \times \mathbb{R}^M$ be a subspace where all feasible solutions of eqs. (61-62) are contained in and let

$$\sup_{(y,x)\in U} \rho(\mathsf{D}_x^2 f) = \bar{\rho} < \infty. \tag{65}$$

Then, if the measurement noise σ_x is small enough, eq. (50) can be solved for $\dot{x}$ in the entire solution space. At this point, existence and uniqueness of a solution of eqs. (54-56) is subject to the same considerations as in the case of functions f that are linear with respect to x.
